# Supplementary material for: Surface Microbiome Profiling of Dental Elevators Using Third‐Generation Sequencing: Implications for Infection Control in Dental Practice
Source: Microbiologyopen. 2025 Dec 8;14(6):e70178. doi: 10.1002/mbo3.70178 (PMC12685768; doi:10.1002/mbo3.70178)
Supplement: Supplementary file 1 — customer_backup. [file MBO3-14-e70178-s001.zip › customer_backup/customer_backup/function_analysis/BugBase/treat/otu_contributions/class/taxa_legend.pdf]

Clostridia
Chloroplast
Betaproteobacteria
Bacteroidia
Bacilli
Alphaproteobacteria
Actinobacteria
Other
Thermoleophilia
Acidimicrobiia
TM7−3
Synergistia
Spirochaetes
Oscillatoriophycideae
Gammaproteobacteria
Fusobacteriia
Erysipelotrichi
Epsilonproteobacteria
Deltaproteobacteria
Coriobacteriia
